# Comparison of Chitosan Nanoparticles and Soluplus Micelles to Optimize the Bioactivity of *Posidonia oceanica* Extract on Human Neuroblastoma Cell Migration

**DOI:** 10.3390/pharmaceutics11120655

**Published:** 2019-12-06

**Authors:** Vieri Piazzini, Marzia Vasarri, Donatella Degl’Innocenti, Asia Guastini, Emanuela Barletta, Maria Cristina Salvatici, Maria Camilla Bergonzi

**Affiliations:** 1 Department of Chemistry, University of Florence, Via Ugo Schiff 6, 50019 Sesto Fiorentino, Italy; vieri.piazzini@unifi.it (V.P.); asia.guastini@stud.unifi.it (A.G.); 2 Department of Experimental and Clinical Biomedical Sciences “Mario Serio”, University of Florence, Viale Morgagni 50, 50134 Florence, Italy; marzia.vasarri@unifi.it (M.V.); emanuela.barletta@unifi.it (E.B.); 3 ICCOM—Centro di Microscopie Elettroniche “Laura Bonzi”, CNR, 50019 Sesto Fiorentino, Italy; salvatici@ceme.fi.cnr.it

**Keywords:** *Posidonia oceanica*, nanoparticles, polymeric micelles, SH-SY5Y cell migration, wound healing assay

## Abstract

*Posidonia oceanica* (L.) Delile is a marine plant endemic of Mediterranean Sea endowed with interesting bioactivities. The hydroalcholic extract of *P. oceanica* leaves (POE), rich in polyphenols and carbohydrates, has been shown to inhibit human cancer cell migration. Neuroblastoma is a common childhood extracranial solid tumor with high rate of invasiveness. Novel therapeutics loaded into nanocarriers may be used to target the migratory and metastatic ability of neuroblastoma. Our goal was to improve both the aqueous solubility of POE and its inhibitory effect on cancer cell migration. Methods: Chitosan nanoparticles (NP) and Soluplus polymeric micelles (PM) loaded with POE have been developed. Nanoformulations were chemically and physically defined and characterized. In vitro release studies were also performed. Finally, the inhibitory effect of both nanoformulations was tested on SH-SY5Y cell migration by wound healing assay and compared to that of unformulated POE. Results: Both nanoformulations showed excellent physical and chemical stability during storage, and enhanced the solubility of POE. PM-POE improved the inhibitory effect of POE on cell migration probably due to the high encapsulation efficiency and the prolonged release of the extract. Conclusions: For the first time, a phytocomplex of marine origin, i.e., *P. oceanica* extract, has enhanced in terms of acqueous solubility and bioactivity once encapsulated inside nanomicelles.

## 1. Introduction

*Posidonia oceanica* (L.) Delile is a marine angiosperm belonging to Posidoniaceae family endemic of the Mediterranean Sea forming expanse underwater meadows of considerable importance for marine ecosystems [1]. The decoction of *P. oceanica* leaves has been dated to ancient Egypt; but more recently, it has been documented to be used by villagers of the sea coast of Western Anatolia as a traditional natural remedy for diabetes, hypertension, and for its antiprotozoal activity [2,3]. In addition, *P. oceanica* has proved to be a promising reservoir of bioactive compounds with antibacterial and antimycotic properties [4]. Over the years, *P. oceanica* has gained a growing interest for its potential benefits on healthcare, mostly related to the antioxidant and antiradical action of its phenolic component. Recently, a study on *P. oceanica* extract highlighted its biological activity even in the dermatological field. In fact, *P. oceanica* has proved to be an efficient anti-aging agent by improving fibroblast activity and collagen production [5]. Moreover, the hydroalcoholic extract of *P. oceanica* (POE) was found to prevent human cancer cell migration with non-toxic mechanism of action. Specifically, the *P. oceanica* phytocomplex has been proven to reduce the motility of human fibrosarcoma cells and the activity of metalloproteases (MMP-2/9) through the activation of a transient autophagic process without any detectable effect on cell viability [6,7]. The anti-inflammatory mechanism of *P. oceanica* phytocomplex was recently elucidated [8].

Neuroblastoma is a common childhood extracranial solid tumor with high mortality originating from the sympathetic nervous system. It represents about 10% of solid tumors and occurs in very young children with an average age of 17 months at diagnosis. The clinical picture of neuroblastoma is very variable and depends on the stage and location of the tumor [9]. In the clinical field, various anti-cancer drugs and therapies are used to prevent the high proliferation of neuroblastoma, including surgery, chemotherapy, immunotherapy, radiotherapy, myeloablative treatment, and retinoids therapy [10]. Despite this, high-stage neuroblastoma presents a poor prognosis with extremely low overall survival. Therefore, the search for novel therapeutics is important in the case of pediatric malignancies to improve patient survival by reducing high toxicity associated with anticancer drugs. Over decades, crude extracts derived from medicinal plants are of great interest for scientific research due to their natural origin and their interesting bioactive compounds which can act synergistically in the prevention or treatment of various human diseases. Furthermore, innovative strategies, like nanotechnology, have achieved great results toward ameliorating cancer therapeutics. The use of new therapeutics delivery system, as nanocarriers, may improve efficacy and decrease systemic toxicity during treatment of malignancies compared to the use of “free” drugs [11,12,13]. Among the varieties of nanoformulations known in the literature, nanoparticles and polymeric micelles are of great interest for pharmacological applications.

In particular, chitosan is one of the polymeric constituents most used in the formulation of nanoparticles, due to its advantageous characteristics and interesting biological activities. It is biocompatible, biodegradable, and free of toxicity. It is a versatile compound, suitable for various routes of administration and multifunctional due to the possibility of functionalizing the molecule to obtain specific targeting. Thanks to its qualities, chitosan is used as nanocarrier of various types of active ingredients: proteins, antibodies, genes, hormones, drugs, but also natural molecules [14]. Chitosan nanoparticles for plant extracts have also been described, such as the *Nigella sativa* L. aqueous extract or the cherry extract from *Prunus avium* L. [15,16].

Regarding polymeric micelles, they are colloidal association systems consisting of blocks of amphiphilic co-polymers, formed by a hydrophilic (e.g., PEG, PVP) and a lipophilic portion (e.g., polyesters, polyanhydrides, and polyamino acids) [17].

Soluplus is a tri-block copolymer consisting of polyvinylcaprolactam-polyvinylacetate-polyethylene glycol. PEG is the hydrophilic portion and polyvinylcaprolactam-polyvinylacetate moieties are arranged in the hydrophobic core. The polymeric micelles have the possibility of incorporating functionality in both core and shell regions: the hydrophobic molecules in the core, less hydrophobic molecules in the core, but near the hydrophilic moiety [18].

Soluplus is biodegradable, and it has a low CMC (7.6 mg/L), which gives its micelles high stability even after dilution [19,20]. Soluplus micelles have been applied to delivery natural and chemical compounds. For example, it has been seen that the use of Soluplus micelles significantly improves the solubility of silymarin, extracted from the fruits of *Silybum marianum* (L.) Gaertn. (Asteraceae), increasing its solubility and its intestinal permeability [21]. Other applications described the use of Soluplus for doxorubicin delivery in the treatment of resistant tumors [20] or to increase acyclovir permeability across the cornea and sclera [22] or even to enhance oral bioavailability and hypouricemic activity of scopoletin [23]. Numerous other applications are described in the literature [24,25,26,27].

Considering the high migratory and metastatic capacity of neuroblastoma, it is possible to exploit new therapies loaded in nanocarriers to improve the drug efficacy in order to counteract these specific neuroblastoma abilities. Nanoformulations can also be used for the delivery of molecules of natural origin or phytocomplexes to optimize the effectiveness of herbal medicines [13,20,27]. In this perspective, the possibility of using the whole crude extract carried by nanocarriers leads to having better biological efficacy due to the synergistic action of the bioactive compounds of the phytocomplex with respect to the activity of the single compounds.

In this work, we therefore studied—for the first time—the anti-migratory ability of POE loaded in nanoformulations on the human neuroblastoma cell line SH-SY5Y. Our goal was to improve both the aqueous solubility of the POE and its inhibitory effect on cancer cell migration, providing a sustained and prolonged release. For this purpose, we developed and compared two types of POE-loaded nanocarriers, such as chitosan nanoparticles (NP-POE) and Soluplus polymeric micelles (PM-POE), usually applied to the delivery of single compounds. This study aims to develop biocompatible, biodegradable and easy to prepare carriers and to extend their application to carry a phytocomplex, containing molecules with different polarity. Given the chitosan characteristics, this polymer has already been applied to the formulation of nanoparticles for the delivery of polar extracts [15,16]. Polymeric micelles are easy to prepare, stable, biocompatible, and suitable for compounds with different polarity. The authors developed mixed polymeric micelles of Soluplus/TPGS-Vit. E for the formulation of silymarin [21].

The nanoformulations were chemically and physically characterized in terms of size, homogeneity, ζ−potential, morphology, encapsulation efficiency, and storage stability. In vitro release studies were also performed. Finally, the inhibitory effect of both NP-POE and PM-POE on SH-SY5Y cell migration was evaluated by the wound healing assay and compared to that of unformulated POE.

## 2. Materials and Methods

### 2.1. Materials

Sigma-Aldrich (Milan, Italy) provided all chemicals, and analytical grade and HPLC grade solvents. Chitosan low molecular weight (Sigma-Aldrich, Milan, Italy; cat no. 448869, mol wt. 50,000–190,000 Da, viscosity 20–300 cP, 1 wt % in 1% acetic acid, 25 °C, Brookfield). Soluplus was a gift of BASF (Ludwigshafen, Germany) with the support of BASF Italia, BTC Chemical Distribution Unit (Cesano Maderno, Monza e Brianza, Italy). Distilled water was obtained from a Simplicity^®^ UV Water Purification System, Merck Millipore (Darmstadt, Germany). Phosphotungstic acid (PTA) was from Electron Microscopy Sciences (Hatfield, PA, USA). Dialysis kit was from Spectrum Laboratories, Inc. (Breda, The Netherlands). Dulbecco’s modified Eagle’s medium (DMEM), Ham’s F-12 nutrient mixture, fetal bovine serum (FBS), L-glutamine, penicillin and streptomycin, 1-(4,5-dimethylthiazol-2-yl)-3,5-diphenyl formazan (MTT) were purchased from Sigma Aldrich-Merck (Saint Louis, MO, USA). Disposable plastics were from Sarstedt (Nümbrecht, Germany).

### 2.2. P. oceanica Extract (POE) Preparation

The leaves of *P. oceanica* were extracted as previously described [6]. Briefly, 10 mL of EtOH/H₂O (70:30 *v/v*) per gram of dried and minced *P. oceanica* leaves were left to shake overnight at 37 °C. Hydrophobic compounds were removed from the water-ethanol extraction by repeated shaking in *n*-hexane (1:1), whereas the hydrophilic fraction, recovered in the lower phase, was dispensed in 1 mL aliquots and then dried. Single batch of *P. oceanica* extract was dissolved in 0.5 mL of EtOH/H₂O (70:30 *v/v*) before to use and hereafter referred to as POE. Freshly-dissolved POE was characterized for total polyphenols and carbohydrates content and antioxidant and radical scavenging activities, according to previously described methods [6,7]. Drug: extract ratio (D.E.R) was 8:1.

### 2.3. HPLC-DAD Analytical Method

POE analyses were performed with an HP 1100 HPLC (Agilent Technologies, Santa Clara, CA, USA) equipped with DAD detector and a Luna Omega Polar (150 × 3 mm, 5 µm) (all from Agilent Technologies) RP-C18 analytical column. The software was HP 9000 (Agilent Technologies). The mobile phase consisted of (A) formic acid/water pH 3.2 and (B) acetonitrile. The following gradient profile was applied: 0–2 min 95% A and 5% B; 2–8 min 75% A and 25% B; 8–10 min 70% A and 30% B; 10–12 min 70% A and 30% B; 12–16 min 60% A and 40% B; 16–18 min 40% A and 60% B; 18–22 min 5% A and 95% B; 22–23 min 5% A and 95% B; 23–25 min 95% A and 5% B. The flow rate was 0.5 mL/min, injection volume 10 μL, and the temperature 25 °C. The calibration curve was prepared using a standard solution of catechin (0.435 mg/mL) diluted 10, 20, 50, 100, 200, and 500 times. The concentration absorption relationship was above than 0.9996. The chromatographic profile of POE polyphenols was acquired at 260 nm and the total polyphenol content was expressed as catechin, using the external standard method.

### 2.4. Preparation of Chitosan Nanoparticles (NP and NP-POE)

NP-POE were prepared using the ionotropic gelation method reported in literature [28,29], modified for the optimization of our formulation. A solution (2 mg/mL) of chitosan (CS) in 1% acetic acid was prepared and kept under magnetic stirring for 24 h, then filtered with a 0.45 μm filter membrane. Tripolyphosphate (TPP) water solution (2 mg/mL) was also prepared and 2 mL of this solution was added to 4 mL of CS solution to prepare empty nanoparticles. The resultant mixture was kept under magnetic stirring for 30 min.

To prepare NP-POE, 4 mL of POE hydroalcoholic solution (5 mg/mL in EtOH/H₂O 70:30 *v/v*) were added to 4 mL of CS solution (2 mg/mL), then 1.5 mL of TPP solution (2 mg/mL) were added dropwise. The mixture was stirred (500 rpm) at room temperature for 30 min, followed by 15 min of sonication in the ultrasonic bath. The final concentrations of POE, CS, and TPP were 2.11 mg/mL, 0.84 mg/mL, and 0.32 mg/mL, respectively.

### 2.5. Preparation of Soluplus Polymeric Micelles (PM and PM-POE)

PM-POE were prepared by the thin film method [17,30,31]. In brief, 250 mg of Soluplus, and 10.55 mg of POE were dissolved in 20 mL of a mixture EtOH/H_2_O (70:30 *v/v*). Then, the solvents were evaporated under vacuum at 30 °C until the formation of a thin film. Finally, the film was hydrated with 5 mL of distilled water under sonication for 5 min followed by 20 min of magnetic stirring at 200 rpm. The final concentration of POE was 2.11 mg/mL. Empty micelles were prepared with the same method.

### 2.6. Physical Characterization by Dinamic Light Scattering (DLS)

Hydrodynamic diameter, size distribution and ζ-potential were measured by Dinamic Light Scattering (DLS), using a Zsizer Nanoseries ZS90 (Malvern Instruments, Worcestershire, UK) outfitted with a JDS Uniphase 22 mW *He*-*Ne* laser operating at 632.8 nm, an optical fiber-based detector, a digital LV/LSE-5003 correlator and a temperature controller (Julabo water-bath) set at 25 °C. Time correlation functions were analysed by the Cumulant method, to obtain the hydrodynamic diameter of the vesicles (Z-average) and the particle size distribution (polydispersity index, PdI) using the ALV-600 software V.3.X provided by Malvern. ζ-potential, instead, was calculated from the electrophoretic mobility, applying the Helmholtz–Smoluchowski equation using the same instrument. The samples were opportunely diluted in distilled water and an average of three measurements at stationary level was taken. A Haake temperature controller kept the temperature constant at 25 °C. The analyses were performed in triplicate.

### 2.7. Morphological Characterization by Transmission Electron Microscopy (TEM)

The morphological characterization of nanoformulations was performed by TEM CM12 (Philips, The Netherlands) equipped with an OLYMPUS Megaview G2 camera at accelerating voltage of 80 keV. Before the analyses, the samples were diluted in distilled water and placed onto a 200-mesh copper grid coated with carbon. Most of the sample was blotted from the grid with filter paper to form a thin film. After the adhesion of formulation, 5 μL of phosphotungstic acid solution (1% *w/v* in sterile water, Electron Microscopy Sciences, Hatfield, PA, USA) were dropped onto the grid as a staining medium and the excess solution was removed with filter paper. Samples were dried for 3 min, after which they were examined with the electron microscope [32].

### 2.8. Encapsulation Efficiency (EE%)

The encapsulation efficiency of the NP-POE was calculated employing the indirect method, as reported in the literature [33]. In brief, the NP-POE were centrifuged at 18000 rpm (Ultracentrifuge Mikro 22, Hettick, Kirchlengern, Germany) for 30 min at 4 °C. The supernatant, containing the non-encapsulated extract, was analyzed by HPLC analysis. POE encapsulation efficiency was calculated according to Equation (1)

(1) $$EE=\frac{Total\mathrm{POE}-Free\mathrm{POE}}{TotalP\mathrm{OE}}\times 100$$

In the case of PM-POE, the dialysis bag method was applied to remove non-encapsulated POE. The bag (cellulose membranes, MWCO 3.5–5 kD, Spectrum Laboratories, Inc., Breda, The Netherlands) was kept in 1 L of distilled water for 30 min at room temperature with continuing stirring at 150 rpm. Then, POE retained in the PM was quantified after dilution with ethanol and sonication for 30 min in an ultrasonic bath. The resulted mixture was analysed by HPLC after centrifugation for 10 min at 14000 rpm [34,35,36]. The Equation (2) was applied to EE% determination.

(2) $$EE=\frac{mgPOEencapsulated}{TotalPOE}\times 100$$

### 2.9. Stability Studies

Stability studies were conducted over 3 months. NP-POE and PM-POE were stored at 4 °C, in the test tubes coated with aluminium foils. The particle size, PdI, ζ-potential and encapsulation efficiency were investigated at regular intervals to assess the chemical and physical stability of the samples.

### 2.10. In Vitro POE Release from NP-POE and PM-POE

The release of POE from NP-POE and PM-POE were studied using the dialysis bag method (cellulose membranes, MWCO 3.5–5 kD, Spectrum Laboratories, Inc., Breda, The Netherlands). In order to mimic the sink condition, as release medium a PBS solution at pH 7.4 was used. Each formulation (2 mL) was introduced into the dialysis membrane and placed in the release medium (200 mL) at 37 °C, under magnetic stirring. The dissolution medium was 0.01 M PBS (pH 7.4, NaCl 0.138 M, KCl 0.0027 M). At different time points, 1 mL of the release medium was taken and replaced with the same volume of PBS to maintain the sink condition [21,37]. The experiment was conducted for 24 h and 72 h for NP-POE and MP-POE, respectively. The amount of released extract was quantified by HPLC. A hydroalcoholic solution of POE (2 mg/mL) was used as a control. All samples were in a sink condition with the same amount of POE. The released extract in the dissolution media was quantified by HPLC [33].

### 2.11. Cell Culture and Culture Conditions

The SH-SY5Y human neuroblastoma cell line, purchased from American Type Culture Collection (ATCC^®^, Manassas, VA, USA), were grown in a 1:1 mixture of Ham’s F12 and DMEM supplemented with 2 mM l-glutamine, 100 μg/mL streptomycin, 100 U/mL penicillin and 10% FBS, at 37 °C in a humidified atmosphere of 5% CO_2_. Once cells reached 70% to 80% confluence, they were detached by trypsinization (0.25% trypsin, 0.5 mM EDTA solution) and propagated after appropriate dilutions. SH-SY5Y experiments were performed in serum-free medium (starvation medium) after exposure to unformulated POE and POE-loaded nanocarriers, as NP-POE and PM-POE, opportunely diluted in culture medium to obtain 3 μg/mL final concentration of POE. Untreated cells were used as control.

### 2.12. Cell Viability Assay

Cell viability was assessed using the colorimetric MTT activity assay. SH-SY5Y cells were seeded in a 96-well plate (5 × 10^3^ cells/well) in complete medium overnight. Then, cells were treated with POE, NP-POE, and PM-POE in starvation medium for 24 h. Culture medium was removed and adherent cells were washed with PBS. Subsequently, 100 μL/well of 0.5 mg/mL MTT solution were added and incubated in the dark at 37 °C for 1 h. Next, cells were washed with PBS and lysed in 80 μL/well of lysis buffer (20% (*w*/*v*) sodium dodecyl sulfate (SDS) in 50% (*v*/*v*) *N*,*N*-dimethylformamide). Absorbance values were measured at 595 nm using iMARK microplate reader (Bio-Rad, Philadelphia, PA, USA). Data on relative cell viability were expressed in terms of percentage of the untreated cells.

### 2.13. Wound Healing Assay

The wound healing assay [6,7] was used to test SH-SY5Y cell migration. Cells were plated in 6-well plate at 5 × 10^5^ cells/well density in complete medium. Once cells reached confluence, a longitudinal scratch was performed through the cell monolayer using a 200 μL sterile plastic tip. Plates were then washed three times with PBS to remove non-adherent cells. Fresh starvation medium containing POE, NP-POE, PM-POE, or empty nanoformulations at appropriate dilutions was added. Cell-free area was observed under phase contrast microscopy and images were captured at 0, 5, 7, and 24 h after wounding using a Nikon TS-100 microscope equipped with a digital acquisition system (Nikon Digital Sight DS Fi-1, Nikon, Minato-ku, Tokyo, Japan). Marked edges along each wound were used to measure cell migration considering the horizontal distance between the initial scratch and the scratch following migration.

### 2.14. Statistical Analysis and Graphics Preparation

The experiments were repeated three times and results were expressed as a mean ± standard deviation, after centralizing mean as a normalization strategy between replicated experiments. The statistical analysis of cell assay was performed with Tukey’s test. Furthermore, the graphs were drawn using LibreOffice Calc. Panels were assembled with LibreOffice Impress and adapted with Gimp 2.8.

## 3. Results and Discussion

### 3.1. Preparation and Characterization of POE-Nanoformulations

#### 3.1.1. NP and NP-POE Preparation

POE has been characterized for the total content of polyphenols and carbohydrates and for its antioxidant and radical scavenging activities as described in previous works [6,7,8] and reported in Table S1 of Supplementary material.

**Figure 1:**
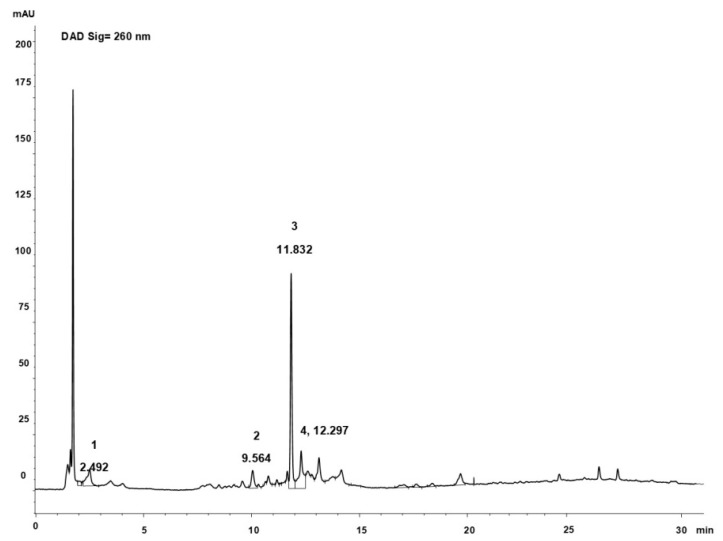
Chromatographic profile of POE (260 nm). 1: gallic acid; 2: chlorogenic acid; 3: catechin; 4: ferulic acid.

The presence of polyphenols was also confirmed by the HPLC analysis of extract [6]. The percentage of polyphenols was 10% of dried extract of *P. oceanica*, the main peak at 11.83 min is catechin, other compounds identified by UV–vis spectrum and by comparison with the standards are reported in the Figure 1, and they are in agreement with previous findings [6].

For the preparation of the empty NP various concentrations and ratio of CS and TPP solutions have been considered. The best system in terms of size PdI and ζ-potential (Table 1) was obtained by reacting 4 mL of the CS solution (2 mg/mL) with 2 mL of the TPP solution (2 mg/mL). Our results are in agreement with previous studies performed with similar conditions [28,29,38].

The incorporation of the extract into the NP was carried out by adding 4 mL of POE hydroalcoholic solution (5 mg/mL) to the CS solution, before adding the TPP solution. In the presence of the extract the amount of the TPP solution was decreased to 1.5 mL respect to the preparation of empty NP, to optimize size and PdI of the sample.

**Figure 2:**
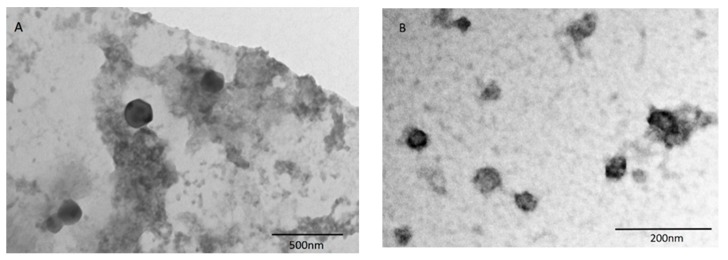
(**A**) TEM image of POE-loaded nanoparticles (NP-POE); (**B**) TEM image of POE-loaded Soluplus polymeric nanomicelles (PM-POE).

Thus, the final concentrations of POE, CS, and TPP were 2.11 mg/mL, 0.84 mg/mL, and 0.32 mg/mL, respectively. The NP are homogeneous, with a positive ζ-potential, due to the presence of chitosan. The loading of the extract inside the NP induces an increase in the size, but the system still remains useful for pharmaceutical administration. As reported in the literature, after drug loading into chitosan nanoparticles, the particle size becomes bigger [39,40]. A possible reason for this phenomenon is that the loaded drug (or extract in this case) reduces the cohesive force between chitosan and tripolyphosphate [41]. TEM analysis shows spherical and not aggregated particles (Figure 2A).

In the case of NP-POE, the encapsulation efficiency was calculated by the indirect method [33], as reported in the experimental section, because the direct method employs drastic conditions [38] which can alter the stability of the extract. The EE% value is 10.63% ± 0.71. This value is not high, but it corresponds to a final POE concentration of 2.11 mg/mL, with 0.22 mg/mL encapsulated into the NP. This represents a remarkable improvement of the aqueous solubility of the extract, that is completely insoluble. This aspect was also confirmed by the change of the color of the mixture, that from colorless becomes light yellow in the presence of nanoparticles. Similar values of EE% were reported in literature for analogous nanoparticles of chitosan carrying natural substances such as eugenol and carvacrol. In the case of eugenol, the EE value ranges from 2% to 29% increasing the initial eugenol content respect to chitosan from 1:0.25 to 1:1.25 *w*/*w*. In the case of carvacrol, the EE% ranges from 13% to 31% with increasing initial carvacrol content [28,38].

Our research represents one of the few studies in which an attempt was made to formulate an extract rather than a single compound. In the literature, there are few examples of nanoformulations of extracts using nanoparticles, such as gelatin NP of *Centella asiatica* and Cardamono extracts [42,43] and chitosan NP of *Nigella sativa* and cherry extracts [15,16]. The nanoparticles are not easy to prepare for extract delivery, due to the presence of various compounds with different polarity, but the application of nanotechnology to extract is of great interest in the phytotherapy field given the remarkable benefits that traditional medicine attributes to the synergistic action of the bioactive compounds present in phytocomplexes.

#### 3.1.2. PM and PM-POE

Soluplus micelles were prepared using the “thin film hydration” technique [17,23,31]. Soluplus is a tri-block copolymer consisting of polyvinylcaprolactam–polyvinylacetate–polyethylene glycol. PEG is the hydrophilic portion and polyvinylcaprolactam–polyvinylacetate moiety are arranged in the hydrophobic core. PM have the possibility of incorporating functionality in both core and shell regions: the hydrophobic molecules of POE in the core, less hydrophobic molecules in the core, but near the hydrophilic moiety [18].

During the optimization process, the hydroalcoholic solution (EtOH/H_2_O 70:30 *v*/*v*) was selected as the best solvent mixture to solubilize both the extract and the polymer. Micelles of Soluplus (5% *w*/*v*), containing 2.11 mg/mL of extract, have the physical parameters showed in Table 2. In the case of PM-POE, the increase of sizes was not observed, probably for the high extract solubilisation in the PM core–shell structure. Indeed, Soluplus exhibits the capability of solubilizing both hydrophobic and hydrophilic drugs into the core and shell of the micelles. This ability might be attributed to the interactions between the drug and the polymer. For example, the phenolic groups might interact with the terminal −OH and ether oxygen groups in Soluplus and form hydrogen bonds [44].

The morphological characterization of PM-POE is reported in Figure 2B. The micelles appear as spherical with the dimensions consistent with those detected by DLS. The dialysis purification method is usually employed for the determination of the encapsulation efficiency in the case of micelles [45,46] and it has been employed as direct process to determine the EE% of PM-POE. The EE% value is 85.55% ± 2.54, corresponding to 1.81 mg/mL of POE effectively encapsulated.

As evidenced for NP-POE, also PM-POE increased the solubility of the extract but with a higher EE% compared to NP-POE. Furthermore, while in an aqueous solution the extract remains completely undissolved, the micellar solution is able to solubilize about 2 mg/mL of POE and it becomes colored in yellow, proof of a change in solubility of the extract. As reported in literature, the nanomicelles have achieved good results in improving the solubility of extracts, such as the silymarin phytocomplex [21]. For the first time in this work Soluplus nanomicelles have been used to increase the solubility of a phytocomplex of marine origin.

### 3.2. Stability Study

Table 2 and Table 3 reported the physical stability of both POE nanoformulations over 3 months at 4 °C. All physical parameters, named size, PdI and ζ-potential, resulted unchanged for both NP-POE and PM-POE.

Also the chemical stability of the formulated extract before and after the storage was substantially comparable, as confirmed by EE% values. The EE% ranges from 10.63 ± 0.71% to 8.83 ± 0.43% after 90 days, and from 85.55 ± 2.54% to 75.80 ± 2.55% after 90 days, are referred to NP-POE and PM-POE respectively.

The PM-POE stability study well correlate with its in vitro activity, that was maintained until 3 months, as proved by its inhibitory effect on SH-SY5Y cell migration.

### 3.3. In Vitro Release Studies

**Figure 3:**
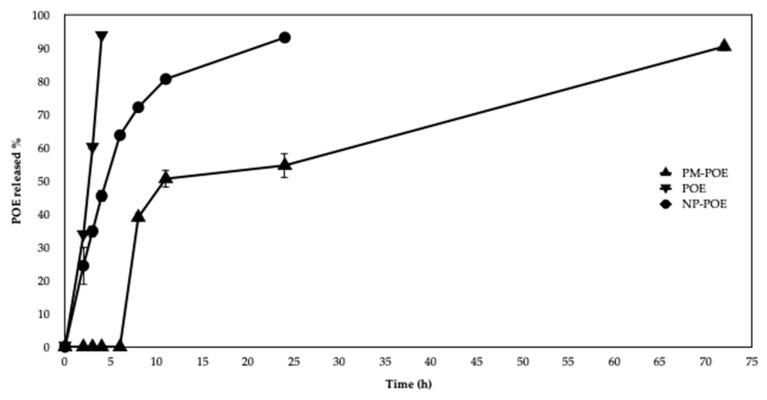
In vitro release profiles of POE from POE solution and POE loaded into nanoformulations (NP-POE and PM-POE).

The release profile of POE solution and POE nanoformulations is shown in Figure 3. In particular, NP-POE released 30% of POE after 3 h, 45% after 4 h and 90% within 24 h of dialysis at 37 °C. The release of the extract by NP-POE is not rapid and immediate as POE solution; the latter in fact already after 3 h reached 60% of release and after 4 h exceeded 90%. NP-POE released the extract in a sustained fashion probably due to the diffusion of the adsorbed extract and its diffusion through the polymeric matrix, mechanisms which govern drug release from chitosan nanoparticles.

As for PM-POE, the release profile was slower and prolonged over time compared to both POE solution and NP-POE, as evidenced in Figure 3. The release of the extract from PM-POE is not immediate but rather delayed, it begins to increase after 5 h, reaching 40% after 8 h, 50% after 12 h, and 90% after 72 h. The obtained results are in agreement with the literature, which refers to a time-delayed release by PM formulations. In fact, it was recently observed that polymeric micelles of Soluplus/P407 release a 6.8% of quercetin in the first 8 h and a 28.75% after 24 h [44]. Evidence of a prolonged lag time in POE release encourages the use of polymeric micelles to optimize POE release.

### 3.4. NP-POE and PM-POE Effect on SHSY5Y Cell Migration

POE and POE-loaded into nanoformulations (NP-POE and PM-POE) were tested on human neuroblastoma SH-SY5Y cell line, at the final concentration of 3 μg/mL according to data previously obtained on POE bioactivities [6,7,8].

**Figure 4:**
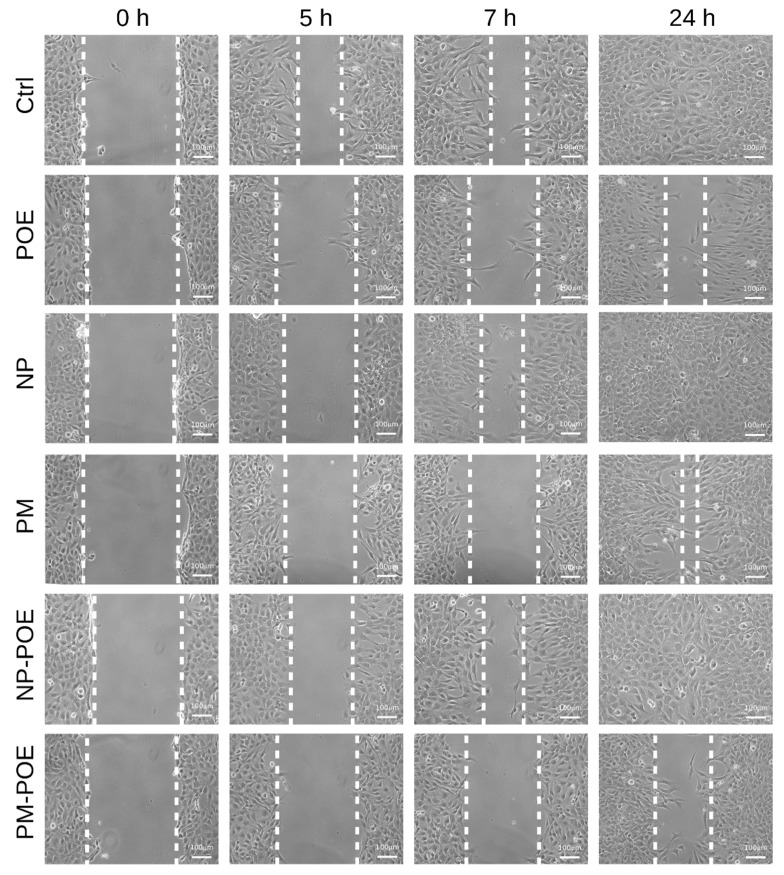
Evaluation of SH-SY5Y cell migration by wound healing assay. Representative image of SH-SY5Y cells, untreated or treated with POE, NP, PM, NP-POE, or PM-POE for 24 h. Scratch closure was monitored over time in cells. The dashed lines mark the edges of the wound area.

**Figure 5:**
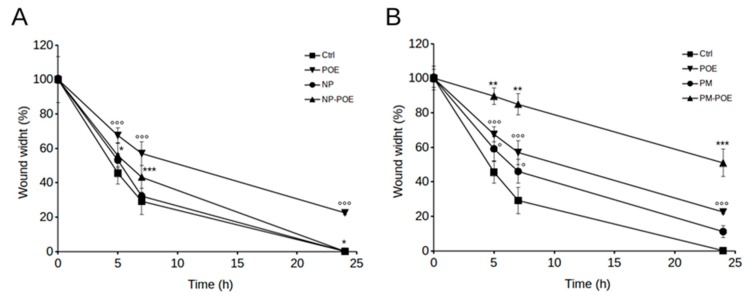
Time course analysis of the scratch closure of SH-SY5Y cells (**A**) treated with NP-POE and (**B**) treated with PM-POE and both with the respective controls. Wound width values were measured considering the horizontal distance between the initial scratch and the scratch following migration at different time points. Data are representative of at least three different experiments. Error bars represent standard deviation. °: *p-*value < 0.05, °°°: *p-*value < 0.001 vs. untreated control cells; *: *p-*value < 0.05, **: *p-*value < 0.01, ***: *p-*value < 0.001 vs. POE treated cells. Tukey’s test.

The effect of POE, NP-POE, and PM-POE on SH-SY5Y cell viability was determined after 24 h treatment by MTT assay, also the effect of empty NP and PM nanocarriers was investigated. In particular, POE had no cytotoxic effects on cell viability as well as cells showed no signs of toxicity in the presence of NP-POE. As for PM-POE, cell viability value was about 80% due to the very low cytotoxicity ascribed to PM as just reported [21] (Figure S1, in Supplementary Material). Therefore, considering that SH-SY5Y cell viability was maintained over 80% up to 24 h of POE, NP-POE, and PM-POE treatments, cell migration was evaluated by the wound healing assay and compared to their vehicles. After wounding cell monolayers, scratched area images were captured at different time points and the distance from the edges was measured. In particular, Figure 4 and Figure 5 show that POE treatment determined a clear reduction of SH-SY5Y cell migration so that as early as 5 h from the initial scratch the wound width was about 70% ± 4%, while the untreated control cells migrated up to about 45 ± 6% of the wound width. The inhibitory effect of POE on cell migration was maintained over time determining a 57 ± 7% of wound width after 7 h of treatment and preventing complete closure at 24 h (22 ± 1% of wound width). Conversely, scratch closure rapidly progressed in untreated control cells after 7 h (29 ± 7%) until complete closure at 24 h.

These results are perfectly in agreement with previously results obtained on the ability of POE to inhibit human fibrosarcoma cancer cell migration [6,7]. Therefore, POE confirmed to be a good candidate for the design of novel therapeutic approaches in phytotherapy. Given the ability of POE to prevent the complete closure of the scratch, we investigated SH-SY5Y cell migration in the presence of NP-POE and PM-POE. The effect of empty NP and PM nanocarriers on cell migration was also monitored over time (Figure 4). As reported in Figure 5A, empty NP and NP-POE showed no effect on SH-SY5Y cell migration leading to a complete closure of the wound at 24 h as the untreated control cells. Differently, PM-POE was able to enhance the inhibitory effect of POE on cell migration (Figure 5B). As early as 5 h PM-POE showed the ability to impair SH-SY5Y cell migration (90 ± 5% of wound width) reducing the wound closure of about 20% compared to 5 h POE treatment.

Comparing the results with those of the in vitro release (Figure 3), the release percentage of POE starts after 5 h and becomes 40% at 8 h, whereas the PM-POE enhanced the inhibitory effect of POE on cell migration as early as after just 5 h. This apparent different behavior of PM in the two in vitro tests can be explained first with the different media and conditions of tests. The in vitro release of POE from PM was done in PBS and using dialysis bag while the wound healing experiment was performed in starvation medium and in direct contact of PM-POE with cells. Furthermore, in the in vitro release assay, POE is released from PM also significantly at shorter times than 8 h. Between 0 h and 8 h there is no release ranging from 0% to 40%, but there are intermediate time points where the release of the extract has already begun. For this reason, it is explained why PM have improved the inhibitory effect of POE on cell migration already after 5 h.

The inhibitory effect of PM-POE on SH-SY5Y cell migration results were clear at 24 h of treatment as PM-POE prevented the complete closure of the wound (50 ± 8% of wound width). The empty PM prevented a total closure of the wound at 24 h maintaining the wound width of approximately 11 ± 4% compared to the initial scratch. This delay in the wound closure with respect to untreated control cells could be ascribed to the reduced cell viability observed after 24 h PM treatment. Considering these results, we obtained that PM-POE are able to improve the inhibitory effect of POE on cell migration. Despite the slight inhibitory effect of the empty PM, at 24 h PM-POE inhibited SH-SY5Y cell migration by 30% more than POE.

The activity of PM-POE was maintained until 3 months confirming the results of the stability study previously reported. PM-POE are amphiphilic carriers able to increase the solubility of both lipophilic and hydrophilic constituents of *P. oceanica* extract, as demonstrated by the high EE% with respect to NP-POE. Moreover, the inhibitory activity of *P. oceanica* on cell migration could be ascribed to the prolonged release of POE from nanomicelles. Therefore, the development of nanoformulations, particularly nanomicelles, could be exploited to improve the traditional application of *P. oceanica* in others chronic diseases, such as diabetes [2] and inflammatory-related diseases [8].

## 4. Conclusions

In this work, we have developed two different POE nanoformulations. Both NP-POE and PM-POE were good candidates for increasing the solubility of *P. oceanica* hydroalcoholic extract, showing good physical and chemical characteristics for parenteral administration and excellent physical and chemical stability during storage at 4 °C for three months. However, only the PM-POE nanoformulation was able to improve the POE inhibitory activity against neuroblastoma cell migration. To date, herbal medicine represents an interesting source for the realization of new drugs. Therefore, the development of adequate systems for the administration of natural compounds, such as nanoformulations, offers an advanced approach to improve the bioavailability and/or optimize the solubility and stability of individual natural compounds or extracts. In this work, for the first time, a phytocomplex of marine origin, i.e., *P. oceanica* extract, has shown an increase in terms of aqueous solubility and bioactivity once encapsulated inside nanomicelles. Therefore, we can assert that Soluplus polymeric nanomicelles are a suitable nanoformulation for the release and the improvement of bioactive properties of phytocomplexes.

## Figures and Tables

**Table 1:** Physical and chemical characterization of both empty and POE loaded NP and MP nanoformulations (Mean ± SD, *n* = 3)

Sample	Average Diameter (nm)	PdI	ζ-Potential (mV)	EE%
NP ^1^	153.70 ± 1.74	0.29 ± 0.02	22.00 ± 0.46	-
PM ^1^	58.25 ± 0.03	0.05 ± 0.01	−5.21 ± 1.10	-
NP-POE ^1^	252.40 ± 5.02	0.24 ± 0.02	19.70 ± 0.76	10.63% ± 0.71
PM-POE ^1^	55.74 ± 0.39	0.08 ± 0.02	−8.47 ± 2.31	85.55 ± 2.54

^1^ NP: chitosan nanoparticles; PM: polymeric micelles of Soluplus; NP-POE: POE-loaded chitosan nanoparticles; PM-POE: POE-loaded polymeric micelles.

**Table 2:** Storage stability test of NP-POE at 4 °C for 3 months (Mean ± SD, *n* = 3)

Days	Average Diameter (nm)	PdI	ζ-Potential (mV)
0	252.40 ± 5.02	0.24 ± 0.02	19.70 ± 0.76
30	276.80 ± 6.34	0.22 ± 0.01	17.80 ± 0.78
60	278.40 ± 5.42	0.22 ± 0.01	18.50 ± 0.64
90	277.50 ± 2.91	0.20 ± 0.01	17.10 ± 0.70

**Table 3:** Storage stability test of PM-POE at 4 °C for 3 months (Mean ± SD, *n* = 3)

Days	Average Diameter (nm)	PdI	ζ-Potential (mV)
0	55.74 ± 0.39	0.08 ± 0.02	−8.47 ± 2.31
30	56.43 ± 6.34	0.09 ± 0.01	−8.52 ± 0.78
60	56.81 ± 0.04	0.12 ± 0.02	−8.65 ± 1.70
90	56.22 ± 0.33	0.10 ± 0.01	−6.53 ± 0.56

## Supplementary Materials

The following are available online at https://www.mdpi.com/1999-4923/11/12/655/s1, Figure S1: SH-SY5Y cell viability. MTT test on cells untreated (control), treated with POE, PM-POE and NP-POE or with vehicles only (PM and NP) for 24 h, Table S1: Total polyphenols and carbohydrates content in POE and its antioxidant and radical scavenging activities.
