# Supplementary material for: Comparison of Chitosan Nanoparticles and Soluplus Micelles to Optimize the Bioactivity of Posidonia oceanica Extract on Human Neuroblastoma Cell Migration
Source: Pharmaceutics. 2019 Dec 6;11(12):655. doi: 10.3390/pharmaceutics11120655 (PMC6955792; doi:10.3390/pharmaceutics11120655)
Supplement: Supplementary file 1 [file pharmaceutics-11-00655-s001.pdf]

pharmaceutics

MDPI

Supplementary Materials: Comparison of Chitosan Nanoparticles and Soluplus Micelles to Optimize the Bioactivity of *Posidonia oceanica* Extract on Human Neuroblastoma Cell Migration

Vieri Piazzini, Marzia Vasarri, Donatella Degl'Innocenti, Asia Guastini, Emanuela Barletta, Maria Cristina Salvatici, and Maria Camilla Bergonzi

Table S1. Total polyphenols and carbohydrates content in POE and its antioxidant and radical scavenging activities. All values (means ± standard deviations, $n = 3$) are expressed in mg/mL of the extract after EtOH/H_2O (70:30 *v*/*v*) resuspension [6–8].

	**Polyphenols**	**Carbohydrates**	**Antioxidant**	**Radical Scavenging**
Method	Folin-Ciocalteau	Phenol/Sulfuric acid	Ferrozine®	DPPH
Reference control	Gallic acid	Glucose	Ascorbic acid	Ascorbic acid
POE	3.6 ± 0.3	7.0 ± 2	0.9 ± 0.2	11.0 ± 0.7

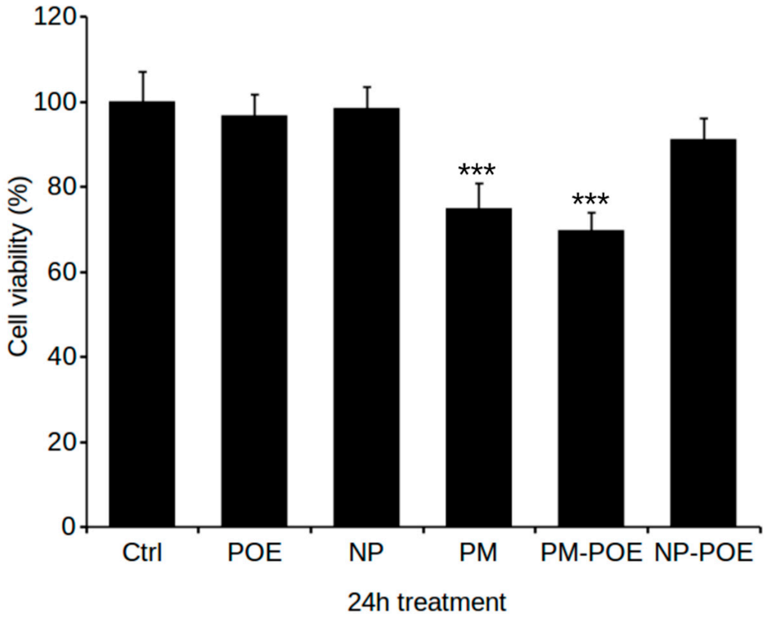

Figure S1. SH-SY5Y cell viability. MTT test on cells untreated (control, Ctrl), treated with POE, PM-POE and NP-POE or with vehicles only (PM and NP) for 24 h. Data were reported as mean ± standard error. ***: *p*-value < 0.001 *vs.* the untreated control cells. Tukey's test, ($n = 3$).

© 2019 by the authors. Submitted for possible open access publication under the terms and conditions of the Creative Commons Attribution (CC BY) license (http://creativecommons.org/licenses/by/4.0/).
